# Grand challenges in parasite epidemiology and ecology

**DOI:** 10.3389/fpara.2022.1034819

**Published:** 2022-10-03

**Authors:** Alyssa E. Barry

**Affiliations:** ^1^ Centre for Innovation in Infectious Disease and Immunology Research (CIIDIR), Institute for Mental and Physical Health and Clinical Translation (IMPACT), Deakin University, Geelong, VIC, Australia; ^2^ Life Sciences Discipline, Burnet Institute, Melbourne, VIC, Australia

**Keywords:** parasite, epidemiology, ecology, omics, One Health, co-infections

## Introduction

Epidemiology and ecology are related disciplines that harness data from collections of organisms together with environmental variables to identify common features and patterns. Never has it been more important to the scientific community and the general-public to understand infectious diseases and all their complexity which is possible using these approaches. Parasitic infectious diseases are no exception, with an enormous diversity of species living in different environments, each of which comprises its own within-species diversity. Many parasites live symbiotically with their hosts, and are important to study from an ecological perspective, however they can also cause significant disease in their host organisms, particularly when a parasite emerges in new hosts or populations.

## Grand challenge 1: One Health - forecasting the next pandemic

‘One Health’, is a relatively new field of research which examines potential disease spread between animals and humans living in shared environments. One Health studies the links between the increasing reliance on animals for food and manufacturing, and the encroachment on wildlife habitats through activities such as deforestation and farming practices and the emergence and spread of zoonotic infections. The COVID-19 pandemic has been a wake-up call for countries to be better prepared for emerging infections that develop into large epidemics or pandemics. Whilst none of the great pandemics have been caused by parasites (Piret and Boivin, 2020), parasitic diseases, such as malaria and helminthiases have historically spread around the world with human migration and technically fit this definition.

Increasingly topical is how parasites interface with their hosts and how these interactions change in context with environmental disturbances. Spill-over events from animals have been the catalyst for many human parasitic infections, including multiple species of malaria parasite, which have originated in non-human primate populations in Africa before emerging and spreading throughout the human population (Duval et al., 2010; Plenderleith et al., 2022). Whilst exhibiting an initial period of animal to human transmission, zoonotic infections pose a great risk of wider spread if they develop the ability to transmit directly between human hosts. *Plasmodium knowlesi* is a good example of an emerging zoonosis that is currently causing significant disease in the human population and has pandemic potential. First identified in humans in Malaysia in 2000, *P. knowlesi* spread from Macaques to humans *via* a mosquito vector (Singh et al., 2004). There has not yet been any evidence of human-to-human transmission, so the chain of infection terminates in humans, however given the presence of this disease across South East Asia (Jeyaprakasam et al., 2020), it is possible that human to human transmission (via the mosquito vector) may eventually evolve. Toxoplasmosis is another parasitic infection that can be spread from animals to humans and is often transmitted between domestic cats and their owners. Whilst toxoplasmosis is mostly a benign infection in humans, it can cause significant pathogenesis in immunocompromised individuals and fetuses (Montoya and Liesenfeld, 2004) and may be associated with miscarriage (Nayeri et al., 2020) and psychiatric disorders (Tyebji et al., 2019). Similar examples of parasitic infections of social and economic importance move between livestock and wildlife, evolving as they go with novel strains posing a major threat to new host populations who have not experienced these infections before.

Understanding the changing ecology and epidemiology of zoonotic parasitic infections and defining modes of transmission can lead to effective strategies to mitigate the further spread of infections. Sharing this knowledge to educate all stakeholders including government, industry, public health, and the community in a timely fashion is ultimately needed to prevent the evolution of human-to-human transmission. In addition, conducting epidemiological surveys and monitoring ecological changes that might impact relevant reservoir populations is of key importance.

## Grand challenge 2: Co-infections - the good and the bad

Co-infection with more than one species of pathogen is common, especially in resource poor settings where access to treatments may be limited. Co-infections with multiple pathogens can have varying impacts on severity and progression of illness, depending on interactions between the species and their host. For example, severe COVID-19 is elicited through an unregulated inflammatory response, therefore it was hypothesised that parasitic infections could potentially protect against severe disease through establishing appropriate immunomodulatory function and immunosuppressive effects (Hays et al., 2020). Soil transmitted helminth infection modulates immune responses and can reduce the severity of other infections including HIV, malaria and schistosomiasis through the reduction of proinflammatory cytokines and may therefore reduce the severity of infectious diseases associated with increased inflammation. Indeed, it was hypothesised that the COVID-19-induced ‘cytokine storm’ might be reduced by pre-existing helminth infection (Hays et al., 2020) and this was verified by epidemiological studies recently (Wolday et al., 2021). In studies of co-infecting parasitic infections, a meta-analysis of 28 studies in Africa showed that co-infection prevalence is high (Afolabi et al., 2021). However, while soil transmitted helminth and schistosome infection was protective against *P. falciparum* malaria, when the analysis was adjusted for potential confounders such as gender, age and socioeconomic status, soil transmitted helminth infection became a risk factor for *P. falciparum* infection (Afolabi et al., 2021). In addition, anaemia was more common in children co-infected with *P. falciparum* and soil transmitted helminths, than with *P. falciparum* malaria alone, whereas *P. falciparum* co-infection with schistosomiasis did not increase the anaemia risk. Other studies investigating co-infections of malaria parasites and soil transmitted helminths have shown contrasting results, and therefore there appears to be no clear consensus as to whether concurrent worm infections are protective or increase risk of malaria symptoms. Similarly, co-infections of schistosomes and soil transmitted helminths such as hookworm and Ascaris are high in tropical regions such as sub-Saharan Africa (Clark et al., 2020). Landscape and spatial epidemiology approaches, which combine prevalence data with potential topographical and geographic risk factors, can produce maps of the predicted prevalence to identify areas where it will be necessary to target disease control efforts. A holistic approach accounting for different parasites and associated infections interacting in a positive or negative way needs to be better understood and integrated into these geospatial approaches to ensure that disease control programs targeting one disease do not result in an increased risk of other diseases, as well as to identify critical determinants of the risks or benefits to hosts.

## Grand challenge 3: Harnessing new technologies and tools

The modern era has provided a seemingly ever-increasing array of Omics approaches which interrogate biological systems such as the transcriptome, proteome, epigenome, metabolome and immunome. Therefore, another important challenge for future parasite ecology and epidemiology research is the combination of traditional sampling frameworks with laboratory analysis of biospecimens to understand various aspects of biology at the population level. Next Generation Sequencing approaches with greater sensitivity and depth allow resolution of low-density infections contaminated with large amounts of host material and can identify genomes or transcriptomes even down to the single cell scale (Howick et al., 2019). The emergence of long read sequencing technologies such as PacBio and Oxford Nanopore additionally offer greater breadth of coverage of genomes, as they can effectively cover highly variable, complex and repetitive genomic regions allowing telomere to telomere chromosome assemblies that short read platforms fail to access (Huddleston et al., 2014). A necessary development alongside these multi-systems biology approaches is advanced analytical approaches to deal with large and complex datasets (Sun and Hu, 2016). Methods to analyse Omics data at both the individual, population level and multi-systems level, combined with advanced biostatistics approaches such as machine learning, mathematical modelling and geospatial approaches will increase precision of associations with ecological and epidemiological variables. In addition, the simplification and portability of molecular analysis platforms, such as the Oxford Nanopore MinION sequencing platform, is bringing the laboratory closer to the field (Maestri et al., 2019), which is particularly relevant to the predominantly field-based disciplines of ecology and epidemiology.

## Call for papers: Research topics and reviews

The Epidemiology and Ecology section of *Frontiers in* *Parasitology *publishes high-quality research across the fields of ecology and epidemiology on parasites of humans, animals and plants. These important areas of parasitology include studies of the parasite life cycle and their environment, and population studies on the distribution and impact of parasites on the health of their hosts. The above challenges are not an exhaustive list – The Frontiers in Parasitology Epidemiology and Ecology Section is interested in studies conducted using cutting edge approaches but also traditional approaches that shed new light on parasite systems. We look forward to receiving both original articles and reviews and have already posted a Research Topic named “Parasite Epidemiology and Ecology using Molecular Approaches”, and another on “Congenital infections”. We have also recently released a call for reviews covering any topic in Parasite Epidemiology and Ecology and look forward to working with the research community to publish your high quality and topical research.

## Author contributions

The author confirms being the sole contributor of this work and has approved it for publication.

## Conflict of interest

The author declares that the research was conducted in the absence of any commercial or financial relationships that could be construed as a potential conflict of interest.

## Publisher’s note

All claims expressed in this article are solely those of the authors and do not necessarily represent those of their affiliated organizations, or those of the publisher, the editors and the reviewers. Any product that may be evaluated in this article, or claim that may be made by its manufacturer, is not guaranteed or endorsed by the publisher.
